# RNA-Seq analysis reveals new gene models and alternative splicing in the fungal pathogen *Fusarium graminearum*

**DOI:** 10.1186/1471-2164-14-21

**Published:** 2013-01-16

**Authors:** Chunzhao Zhao, Cees Waalwijk, Pierre J G M de Wit, Dingzhong Tang, Theo van der Lee

**Affiliations:** 1 Plant Research International, P.O. Box 6708 PB, Wageningen, The Netherlands; 2 Graduate School Experimental Plant Sciences, Wageningen, The Netherlands; 3 State Key Laboratory of Plant Cell and Chromosome Engineering, Institute of Genetics and Developmental Biology, Chinese Academy of Sciences, Beijing, 100101, China; 4 Graduate University of Chinese Academy of Sciences, Beijing, 100049, China; 5 Wageningen University, Laboratory of Phytopathology, P.O. Box 6708 PB, Wageningen, The Netherlands

**Keywords:** *Fusarium graminearum*, RNA-Seq, Alternative splicing, Gene annotation, Novel transcriptionally active regions

## Abstract

**Background:**

The genome of *Fusarium graminearum* has been sequenced and annotated previously, but correct gene annotation remains a challenge. In addition, posttranscriptional regulations, such as alternative splicing and RNA editing, are poorly understood in *F. graminearum*. Here we took advantage of RNA-Seq to improve gene annotations and to identify alternative splicing and RNA editing in *F. graminearum*.

**Results:**

We identified and revised 655 incorrectly predicted gene models, including revisions of intron predictions, intron splice sites and prediction of novel introns. 231 genes were identified with two or more alternative splice variants, mostly due to intron retention. Interestingly, the expression ratios between different transcript isoforms appeared to be developmentally regulated. Surprisingly, no RNA editing was identified in *F. graminearum*. Moreover, 2459 novel transcriptionally active regions (nTARs) were identified and our analysis indicates that many of these could be missed genes. Finally, we identified the 5′ UTR and/or 3′ UTR sequences of 7666 genes. A number of representative novel gene models and alternatively spliced genes were validated by reverse transcription polymerase chain reaction and sequencing of the generated amplicons.

**Conclusions:**

We have developed novel and efficient strategies to identify alternatively spliced genes and incorrect gene models based on RNA-Seq data. Our study identified hundreds of alternatively spliced genes in *F. graminearum* and for the first time indicated that alternative splicing is developmentally regulated in filamentous fungi. In addition, hundreds of incorrect predicted gene models were identified and revised and thousands of nTARs were discovered in our study, which will be helpful for the future genomic and transcriptomic studies in *F. graminearum*.

## Background

*Fusarium graminearum* is an ascomycete that can cause diseases in a variety of agronomically important crops, including Fusarium Head Blight (FHB) on wheat, barley and oat, and stalk rot on corn [1,2]. Infection by *F. graminearum* not only causes severe yield losses but also contaminates seeds with mycotoxins, such as deoxynivalenol (DON) and nivalenol (NIV) [3,4], which are very harmful to humans and animals [5,6]. The infection of crops by *F. graminearum* is still poorly understood, but genome and transcriptome research will enable us to identify genes that are required for pathogenicity and improve our understanding of infection mechanism of *F. graminearum* on its host plants. The genome of *F. graminearum* has been sequenced and currently two different annotations of the same genome assembly are available. One was generated by the Broad Institute [7], and a second one by MIPS [8,9].

The correctness of predicted gene models is extremely important for further comparative and functional genome studies. Gene model predictions performed at the Broad Institute were mainly generated by machine annotation based on a combination of the Calhoun annotation system and the FGENESH program [7]. The MIPS *F. graminearum* database was constructed based on Broad gene calls by integrating several sources and programs, including (i) integration of different gene prediction programs, (ii) comparison of current *F. graminearum* gene models with related *Fusarium* species (*F. oxysporum, F. verticillioides* and *F. solani*) and other *Ascomycetes* including *Neurospora crassa*, and (iii) inclusion of expression sequence tag (EST) data [9]. Compared to the Broad gene set, 1770 gene models were revised and 691 new gene calls were added to MIPS gene set [9]. Although many gene models have been improved by these different approaches, most of them lack experimental support and for species-specific and non-conserved genes the gene model predictions are often incorrect or partially incorrect. In addition, it is difficult to identify novel genes and delineate untranslated regions (UTRs) using traditional bioinformatics tools. To further improve gene model predictions, large-scale transcript information is required.

Genome sequencing and annotation have provided a global view of the genes present in *F. graminearum*, but little is known about their transcriptional and post-transcriptional regulation. In *Homo sapiens*, *Mus musculus*, *Drosophila melanogaster* and *Arabidopsis thaliana*, alternative splicing has been reported to occur in many genes, which enables these organisms to enlarge their proteome diversity by increasing transcript variations in their genome [10-15]. A striking example of alternative splicing is the *Dscam* gene of *D. melanogaster*, which potentially generates more than 38,000 different transcripts [16]. In mammals, alternative splicing plays an important role in developmental processes, such as stem cell self-renewal and differentiation [17-19], development of heart and brain [20-22], and in the response to extracellular stimuli, such as immune cell activation and neuronal depolarization [23,24]. In *A. thaliana*, alternative splicing has been shown to play an important role in its development [25] and in the response to environmental stimuli, such as light, cold and heat treatment [13]. Alternative splicing has also been reported in fungi, including *Cryptococcus neoformans*, *Ustilago maydis*, *Magnaporthe grisea*, *Aspergillus nidulans*, and *F. verticillioides*[26-29]*.* However, so far, alternative splicing has not been reported to occur in *F. graminearum*.

Recently, next-generation sequencing technology (RNA-Seq) has become available as a powerful tool to investigate the transcriptional profiles in many organisms, such as *H. sapiens*, *Saccharomyces cerevisiae*, *A. thaliana*, *Candida albicans* and *C. parapsilosis*[13,30-33]. It has been demonstrated that RNA-Seq data can be efficiently used to improve gene model prediction and to identify novel transcripts [34-36]. In addition, RNA-Seq technology is much more sensitive and efficient than previously used dedicated microarrays to compare gene expression profiles [10]. RNA-Seq data also have been successfully used to identify alternative splicing in genes of different species [11,13,37]. Moreover, RNA-Seq technology has recently been used to identify RNA editing in *H. sapiens*[38].

Previously, we have identified and phenotypically characterized knock out mutant *ebr1* (*Enhanced branch 1*) that shows reduced radial growth and reduced pathogenicity [39]. EBR1 encodes a Gal4-like Zn_2_Cys_6_ transcription factor that is localized in the nucleus during vegetative growth. In order to further unravel the regulatory role of EBR1 in radial growth, we have performed RNA-Seq on wild-type isolate PH-1 (PH-1) and mutant *ebr1* (*ebr1*) to identify differentially expressed genes. In this study, we focused on the use of RNA-Seq data from both PH-1 and *ebr1* to improve gene model predictions, identify novel genes, and search for alternative spicing and RNA editing in *F. graminearum*. The obtained results were validated using RT-PCR and sequencing of the generated products. These analyses have improved numerous gene models and provided a comprehensive insight of RNA splicing in *F. graminearum*.

## Results

### Quality analysis of the RNA-Seq data from *F. graminearum*

**Figure 1:**
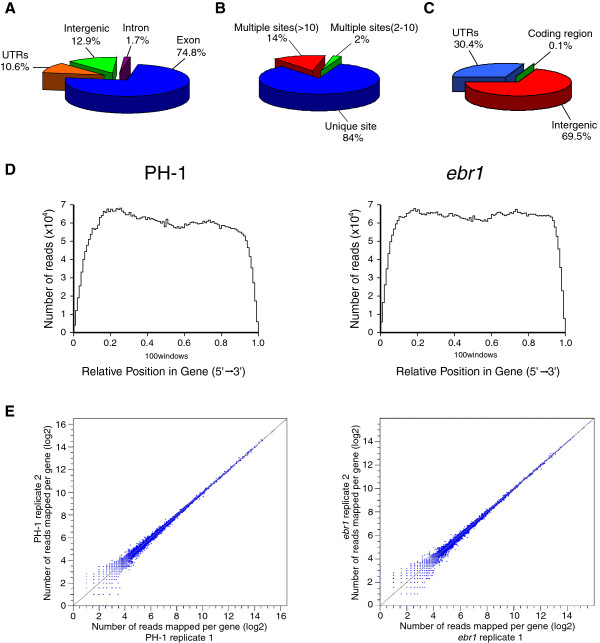
**Statistical and quality control analysis of RNA-Seq data. A.** Read distribution over exons, introns, untranslated regions (UTRs) and intergenic regions. **B.** 84% of all reads matched to unique locations. The remaining 16% of reads matched to multiple locations in the genome of *F. graminearum* (2% matched to 2–10 sites and 14% to more than 10 different locations). **C.** The distribution of all reads matching to multiple locations in intergenic regions, UTRs and coding regions. **D.** The total read coverage along the gene body from 5′ to 3′ end in wt PH-1 and mutant *ebr1*. The genes were divided into 100 equal windows. **E.** Scatter plot analysis of two technical replicates from both wt PH-1 and mutant *ebr1*. Log2 transformed reads number of all predicted genes was used for comparison.

To perform RNA-Seq analysis, RNA was isolated from mycelia of PH-1 and *ebr1* grown in liquid CM medium for 30 h. The isolated RNA was prepared to be sequenced by next generation sequencing technology (Illumina). Of each isolate, two technical replicates were analyzed. In total 12,791,946 reads (90 nucleotides for each read) from PH-1 and 12,928,704 reads from *ebr1* were obtained. Using the genome annotation in the Broad database, transcripts were detected for 76.9% of the 13321 predicted genes in PH-1 and for 81.2% of the genes in *ebr1* (Additional file 1 and Additional file 2). By combing the RNA-Seq data from both PH-1 and *ebr1* we detected the expression of over 84% of the predicted genes. In addition, the combined RNA-Seq data showed that 74.8% of the reads matched to exonic regions, 10.6% to untranslated regions (UTRs), 12.9% to intergenic regions and only 1.7% to intronic regions (Figure 1A). Among all matched reads, 84% matched to unique locations, and 16% to multiple locations in the genome, of which 2% matched to between 2 to 10 different locations, and 14% to more than 10 different locations (Figure 1B). Of all reads that matched to multiple locations, 69.5% matched to intergenic regions, 30.4% to UTRs, and only 0.1% to coding regions (Figure 1C).

To evaluate the quality of RNA-Seq data, several quality control analyses were performed. Firstly, the total coverage of reads from the 5′ to the 3′ end of genes was examined. For both PH-1 and *ebr1* RNA-Seq reads were evenly distributed the exception of the very 5′ and 3′ ends (Figure 1D). In addition, for 54% of the genes in PH-1 and 60% of the genes in *ebr1* the read coverage was more than 90% (Additional file 3: Figure S1). Finally, comparison of the two technical replicates of both PH-1 and *ebr1* clearly showed that the RNA-Seq data are highly reproducible (Figure 1E).

**Figure 2:**
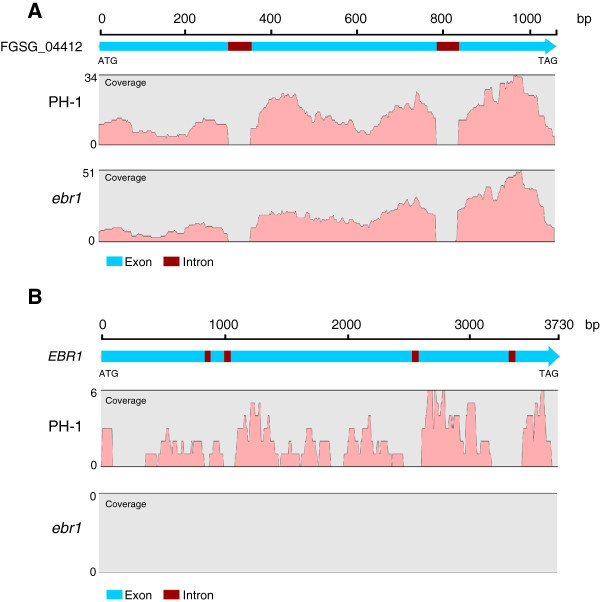
**Visualization of RNA-Seq data in the CLC genomic workbench software. A.** Reads from the RNA-Seq data of wt PH-1 and mutant *ebr1* were mapped to the gene FGSG_04412. 168 reads in PH-1 and 216 reads in *ebr1* uniquely matched to the exon of FGSG_04412. In contrast, no reads matched to the intronic regions. Y-axis represents the coverage number of reads of each nucleotide. **B.** Analysis of the reads matching to the *EBR1* gene in wt PH-1 and mutant *ebr1*, respectively. 75 reads from wt PH-1 RNA-Seq data matched to *EBR1*, whereas no reads from mutant *ebr1* RNA-Seq data matched to *EBR1*.

Matching of the reads to the *F. graminearum* gene database showed that the 98.1% of the matched reads supported the gene models present in the Broad *F. graminearum* database, implying that reads matched to exonic, but not to intronic regions of genes. For example, all 168 reads from PH-1 and all 216 reads from *ebr1* uniquely matched to the exonic regions of gene FGSG_04412, and no reads were found matching to the intronic regions (Figure 2A). Comparing the reads matching to gene FGSG_04412 from PH-1 and *ebr1* showed that the distribution pattern of reads along the gene was very similar (exemplified in Figure 2A). Similar distribution patterns of reads in PH-1 and *ebr1* were also found in other genes. To evaluate the background of non-specific transcripts, we compared the expression of *EBR1* in PH-1 and *ebr1*. In PH-1, 75 reads were found that matched to the coding region of *EBR1*, whereas no reads were found matching to *EBR1* in *ebr1* (Figure 2B). Altogether these results clearly show that the obtained RNA-Seq data are of high quality and form a firm basis for further analysis.

### Strategies to identify incorrect gene models and alternative splicing

We combined the RNA-Seq data of PH-1 and *ebr1* and employed three different strategies to identify incorrect gene models and alternative splicing in *F. graminearum* (Additional file 3: Figure S2A). The first strategy was to identify reads that matched to intronic regions. Reads matching intronic regions originate from either incorrectly annotated or alternatively spliced genes. The second strategy was aimed at predicting transcripts with non-matched or mismatched regions. Of highly expressed genes, the transcripts should be well covered by reads. However, of some transcripts regions not matched by reads or not perfectly matched by reads were identified which points to novel introns or incorrectly predicted introns in these genes. Two examples of this type of transcripts are shown in Additional file 3: Figure S3. In total, 436 possibly incorrectly annotated or alternatively spliced genes were identified by the first strategy and 343 by the second. To further refine incorrect gene models and identify genes with alternative splicing, the TopHat program was applied. This program identifies intron splice sites and has been widely applied to identify incorrect gene annotations and alternative splicing [40]. By applying this program, we obtained 228 putatively new genes. Comparing all these three strategies, we identified 287 genes that were exclusively identified by the first strategy, 243 genes by the second, and 153 genes by the TopHat program. Only 6 genes were identified by all three strategies (Additional file 3: Figure S2B).

Using these three strategies, 842 genes with possibly incorrect gene models or alternative splicing were identified when compared with the Broad *F. graminearum* annotation. We further examined these genes in the MIPS *F. graminearum* database and found that 278 of the identified genes had already been revised (Additional file 4). Subsequently, we manually examined the remaining 564 genes in the CLC software package and classified them into two distinct groups: incorrect gene models and alternatively spliced genes. To distinguish between these two options, we carefully examined reads for the presence of splice sites. Genes that matched reads showing both reference splice site and additional splice site were considered to be the result of alternative splicing; genes that matched reads only showing additional splice site but not reference splice site were grouped into incorrect gene models.

### Identification of incorrect gene models

**Figure 3:**
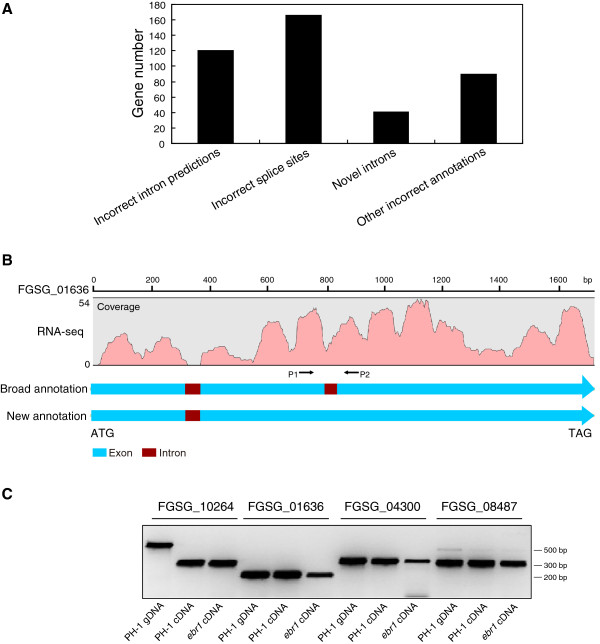
**Identification of incorrect gene models in the *F. graminearum *database. A.** Statistical analysis of the incorrect gene models identified from RNA-Seq data, including incorrect predictions of introns, incorrect intron splice sites, novel introns and other incorrect annotations. **B.** One example of a gene with incorrect intron predictions is shown. Two introns were annotated in gene FGSG_01636 in the Broad *F. graminearum* database, but RNA-Seq data clearly showed that the second intron is absent. **C.** Three genes with incorrect intron predictions were selected for confirmation by RT-PCR. For each gene, one genomic DNA template and two cDNA templates (one is from wt PH-1, one is from mutant *ebr1*) were tested. Primers were designed flanking the intronic regions, as shown in B. Bands with identical size were amplified from both genomic DNA and cDNA template for all three genes, indicating that predicted introns do not exist. The correctly annotated gene FGSG_10264 was used as a control.

377 genes that were incorrectly annotated in the Broad *F. graminearum* database and had not yet been revised in the MIPS *F. graminearum* database were further analyzed. They were divided into four classes: (i) gene models with incorrectly predicted introns, (ii) gene models with incorrect intron splice sites, (iii) gene models with novel introns and (iv) gene models with other incorrect annotations (Figure 3A). In total 119 genes with incorrect intron predictions were identified (Additional file 5). For example, according to the annotation in the Broad *F. graminearum* database, there are two introns in gene FGSG_01636. However, several RNA-Seq reads that matched to the second intron indicated that the proposed second intron does not exist (Figure 3B). For confirmation of this result, primers flanking the supposed second intron were designed and RT-PCR was performed. Genomic DNA isolated from PH-1 and two cDNAs isolated from PH-1 and *ebr1* were used as templates, respectively. RT-PCR confirmed that the proposed second intron in this gene is absent (Figure 3C). Open reading frame inspection showed that the newly proposed gene model is translated into a functional protein without a premature stop. Furthermore, two additional randomly chosen genes, FGSG_04300 and FGSG_08487, were inspected using the same strategy as described for FGSG_01636, and both lacked the predicted introns in the amplified fragments. In contrast, gene FGSG_10264 that was selected as positive control confirmed the predicted intron (Figure 3C).

In addition to incorrect intron predictions, we identified novel introns in 40 genes (Additional file 6). Additional file 3: Figure S4A shows an example of a novel intron identified in gene FGSG_06363. To validate the presence of novel introns, flanking primers for five randomly selected introns were designed and for all of them the presence of the introns was confirmed by RT-PCR (Additional file 3: Figure S4B).

In 164 genes incorrectly predicted splice sites were identified, including incorrect donor and acceptor sites or both; they were manually revised according to our RNA-Seq data (Additional file 7). Additional file 3: Figure S5A shows an example of an incorrectly predicted splice site. Three genes with incorrectly predicted splice sites were randomly selected and were all confirmed by RT-PCR (Additional file 3: Figure S5B). In addition, 88 genes were identified with incorrect gene models of which the correct splice sites could not be assigned yet due to low read coverage or other reasons. Comparison of these genes models with our RNA-Seq data are shown in Additional file 3: Figure S6.

Gene expression analysis showed that for 15% of the predicted genes transcripts were absent in the RNA-Seq data. To determine whether these genes result from incorrect gene calls in databases or were not expressed under the condition tested, we performed a homology search of the predicted proteins using blastP against the NCBI database. As orthologous genes could be identified for 86.5% of these genes (E-value<1E-10), we conclude that these genes are correctly annotated but not or very lowly expressed in liquid CM medium.

### Identification of alternatively spliced genes in *F. graminearum*

**Figure 4:**
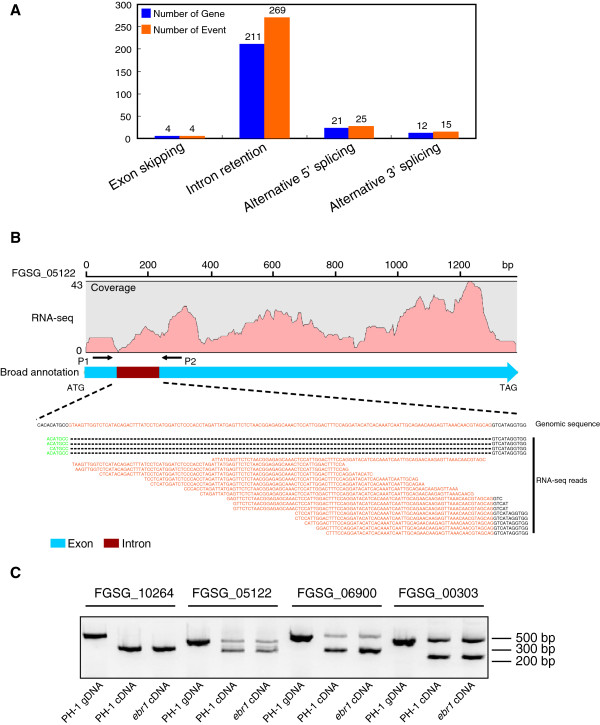
**Identification of alternative splicing. A.** Statistical analysis of alternative splicing identified from RNA-Seq data. Four different types of alternative splicing were identified. Blue bars represent the number of genes that are alternatively spliced. Orange bars represent the total number of alternative splicing events. **B.** FGSG_05122 is an example of a gene showing intron retention. There are four reads representing the intron splice sites (green letters). Meanwhile, several reads uniquely match to the intronic region. Black letters represent exonic region; orange letters represent intronic region. **C.** Three genes with intron retention were selected for confirmation by RT-PCR. Primers were designed flanking the intronic regions. Gene FGSG_10264 was included as a control.

From our RNA-Seq data, 231 genes were identified with alternative splicing, including exon skipping, intron retention, or alternative 5′ and 3′ splice sites (Figure 4A). Most of the alternatively spliced genes involved intron retentions (Additional file 8) of which one example is shown in Figure 4B. In gene FGSG_05122, there are four reads that confirm reference intron splice sites, whereas several reads uniquely matched to a presumed intronic region. To confirm retention of these introns, RT-PCR was performed for three randomly selected genes, and in all cases the predicted intron retentions were confirmed (Figure 4C). Open reading frame analysis of genes with intron retention showed that most of them lead to premature termination of translation (Additional file 8).

**Figure 5:**
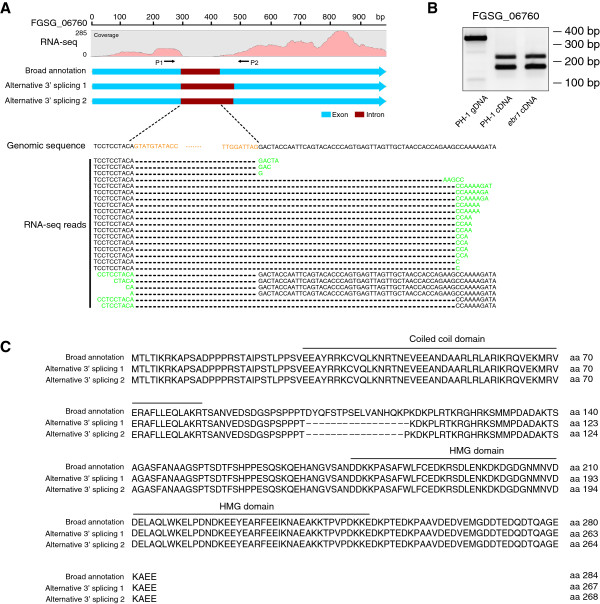
**Example of alternative 3′ splicing. A.** Three different 3′ intron splice sites were identified in the intron of gene FGSG_06760. There are seven reads showing the reference 3′ intron splice site, 16 reads showing alternative 3′ intron splice site 1, and one read showing alternative 3′ intron splice site 2. Black letters represent the exonic region; orange letters represent the intronic region; green letters represent different 3′ intron splice sites. **B.** RT-PCR confirmed the alternative 3′ splicing events in the intron of FGSG_06760. **C.** Protein alignment shows that 16 or 17 amino acids located between coiled coil and HMG domain are lacking in the proteins encoded by the two alternatively spliced transcripts.

In addition to intron retention, we identified 28 genes with alternative 5′ or 3′ splice sites or both (Additional file 9 and Additional file 10). Similar to genes with intron retention, most genes with alternative 5′ and 3′ splice sites led to premature termination of translation. Among all these alternatively spliced introns, we identified three introns with two alternative 3′ splice sites and three with two or three alternative 5′ splice sites. For instance, gene FGSG_06760 that encodes a HMG-box protein with a coiled coil and HMG domain contains two alternative 3′ splice sites according to RNA-Seq data (Figure 5A). The alternative 3′ splice sites were confirmed by RT-PCR (Figure 5B). Compared to the predicted transcript based on the gene model present in the Broad database, the two alternative 3′ splice sites do not lead to premature termination of the transcript. However, the two alternative transcripts lead to proteins lacking 16 amino and 17 amino acids, respectively, that are located between the coiled coil and HMG domain (Figure 5C). Another example is gene FGSG_06124 for which there are four different 5′ splice sites in its second intron (Additional file 3: Figure S7A) that were all confirmed by RT-PCR (Additional file 3: Figure S7B). All alternative transcripts are not prematurely terminated (Additional file 3: Figure S7C). FGSG_06124 encodes a hypothetical protein, with a putative transmembrane and prolipoprotein diacylglyceryl transferase domain. Both domains are present in all four predicted proteins.

Finally, we identified four cases of exon skipping (Additional file 11). FGSG_00786 is an example of a gene with alternative exon skipping (Additional file 3: Figure S8 A) that encodes a serine/threonine-protein kinase srk1 with an S_TKc domain between amino acid (aa) residues 101 and 405 (Additional file 3: Figure S8B). The third exon in FGSG_00786 is sometimes lacking in transcripts as was confirmed by RT-PCR (Additional file 3: Figure S8C), leading to the loss of 17 aa residues in the S_TKc domain.

From above, six genes with alternative splicing were confirmed in both PH-1 and *ebr1* by RT-PCR. We further analyzed all remaining alternatively spliced genes by using RNA-Seq data from PH-1 and *ebr1*, respectively, in the CLC software package. Nearly all of the alternative splicing events can be identified in both PH-1 and *ebr1*. This indicates that disruption of *EBR1* in *F. graminearum* does not affect alternative splicing. To further understand possible roles of all alternatively spliced genes, we functionally categorized them by using the MIPS FunCatDB database. The alternatively spliced genes did not belong to one specific functional class of genes, but were classified in many different categories, of which “proteins with binding function or cofactor requirement” (*P*-value=1.91E-06) and “Protein synthesis” (*P*-value=2.61E-04) prevailed.

### Alternative splicing is developmentally regulated

**Figure 6:**
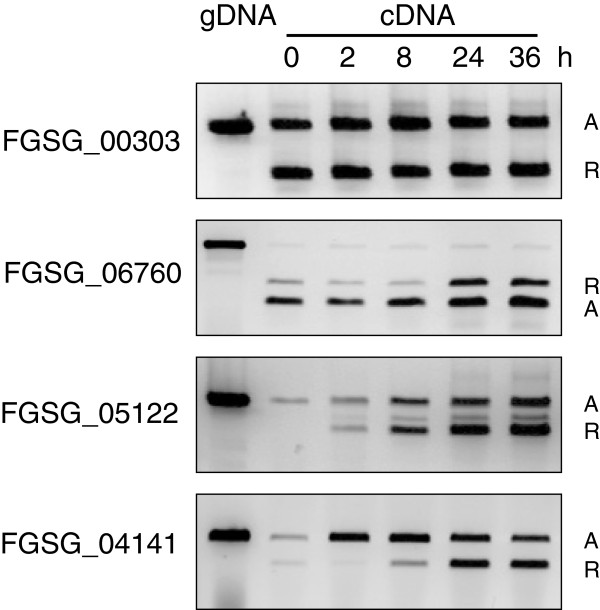
**Alternative splicing is developmentally regulated.** RNA samples were isolated from wt PH-1 isolate at five different time points (0 h, 2 h, 8 h, 24 h, and 36 h after incubation of conidia in liquid CM medium-containing shake cultures). RT-PCRs were performed at the different time points for four alternatively spliced genes (FGSG_00303, FGSG_06760, FGSG_05122 and FGSG_04141). Genomic DNA template was used as control. Capital letter R represents reference transcript isoform. Capital letter A represents alternatively spliced transcript isoform. The experiment was independently performed twice with similar results.

To test whether alternative splicing in *F. graminearum* is developmentally regulated, we performed RT-PCR on four alternatively spliced genes (FGSG_00303, FGSG_06760, FGSG_05122 and FGSG_04141) on PH-1 RNA samples isolated at five different time points (0 h, 2 h, 8 h, 24 h and 36 h after incubation of conidia in liquid CM medium) (Figure 6). In this medium, the macroconidia of *F. graminearum* swell within 2 h, germinate after 3 h and the hyphae elongate and develop into mycelium after 8 h [41]. For gene FGSG_00303 (encoding a transcriptional elongation regulator), the expression ratio between the two transcript isoforms is independent of the growth stage, whereas this ratio (reference isoform/alternative isoform) increased for gene FGSG_06760 (encoding a HMG box protein) after 8 h. The expression of genes FGSG_05122 (encoding a FAD dependent oxidoreductase) and FGSG_04141 (encoding a DNA repair protein) show strong developmental regulation and the expression ratio between the two transcript isoforms changes strongly at different vegetative growth stages. The RT-PCRs for these genes were biologically repeated with similar result. These data suggest that for some of the alternatively spliced genes the expression levels and the ratio between transcripts change during vegetative growth.

### Non-canonical splice sites

**Figure 7:**
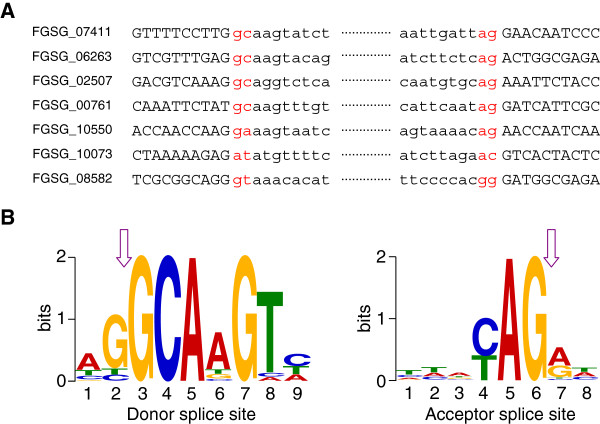
**Non-canonical splice sites identified in *F. graminearum. *A.** Genes with non-canonical splice sites are shown. Capital letters represent exonic sequences, small letters represent intronic sequences. **B.** Analysis of nucleotides preference flanking GC donor splice site and AG acceptor splice site by using the MEME program [42]. Y-axis indicates sequence conservation at each position. The height of symbols in each position represents the relative frequency of each nucleic acid. Arrows indicate splice sites.

Manually inspection of the 842 genes with putative incorrect gene models or alternative splicing resulted in the identification of 28 genes with non-canonical splice sites (Additional file 12). In total the 842 genes contain 2604 introns, of which 98.92% carry canonical GT-AG donor-acceptor sites, 0.77% introns carry GC-AG donor-acceptor sites, and the remaining 0.31% carry other non-canonical donor-acceptor sites, including GT-GG, AC-AC, GG-TA, TA-AG, AT-AC and GA-AG. Seven genes with non-canonical splice sites are shown in Figure 7A and the correctness of all these splice sites was confirmed by Sanger sequencing showing that they were not caused by sequencing errors.

The 20 introns with GC-AG splice sites were analyzed for the presence of conserved flanking nucleotides by using motif comparison tool [42]. AG nucleotides predominantly flank the GC donor site, whereas in the intronic region, AAGT occurs more frequently. The nucleotides flanking the AG acceptor site are less conserved. However, a C or T prevails in the intronic region flanking AG (Figure 7B).

### Identification of novel transcriptionally active and untranslated regions

By mapping RNA-Seq reads against the Broad *F. graminearum* database, 12.9% of the reads matched to intergenic regions, from which 2459 novel transcriptionally active regions (nTARs) were obtained (Additional file 13). To determine whether these nTARs encode proteins, they were blasted against the MIPS *F. graminearum* and Broad *Fusarium* databases. Of these 2459 nTARs, 355 had already been predicted as novel genes in the MIPS *F. graminearum* database, 118 of which show orthologs in either the *F. oxysporum, F. verticillioides* or both. In addition, we identified 74 nTARs that had not yet been annotated in the MIPS *F. graminearum* database but are putatively derived from genes as orthologs were identified in either *F. oxysporum*, *F. verticillioides* or both. In addition, we found 123 nTARs (5%) that contain introns, indicating that they could be real genes (Additional file 13). Additional file 3: Figure S9A shows an example of an nTAR, TU358, which contains three introns. To confirm that the identified nTARs are real, five were selected and confirmed by RT-PCR (Additional file 3: Figure S9B).

The RNA-Seq data also allowed identification of the boundaries of 5′ and 3′ UTRs of genes. For 5951 genes 5′ UTRs and for 6405 genes 3′ UTRs were identified (Additional file 3: Figure S10A, Additional file 14 and Additional file 15). Comparing UTRs identified by RNA-Seq analysis with those present in the annotated genome in the Broad *F. graminearum* database showed some genes with incorrectly predicted UTRs. One example is shown in Additional file 3: Figure S10B where the 3′ UTR prediction in gene FGSG_01403 is different from that predicted by RNA-Seq analysis.

### Screening for RNA editing in *F. graminearum*

In total 695 single nucleotide polymorphisms (SNPs) were identified when comparing RNA-Seq data with the genome sequences by using the CLC software package. All SNPs were manually examined and a large number was identified in stretches of multiple cytosine residues. In addition, many SNPs were identified near intron splice sites and appeared to be caused by misalignment of cDNA to the genomic DNA sequence. Twelve representative SNPs were selected for confirmation by Sanger sequencing of the PCR amplicons obtained from both genomic DNA and cDNA. In four cases the SNPs were not real and due to sequencing errors present in the genomic DNA sequence of PH-1. For the remaining eight SNPs, no differences were observed between cDNA and genomic sequences after re-sequencing suggesting that in the latter cases discrepancies between the RNA-Seq data and the genome sequence could be explained by sequencing errors in the initial RNA-Seq data set. These results suggest that no RNA editing occurs in *F. graminearum* according to our analysis.

## Discussion

In this study we analyzed the transcriptome of *F. graminearum* grown in liquid CM medium by Illumina sequencing to investigate the correctness of predicted gene models present in the annotated Broad *F. graminearum* genome database and to identify the occurrence of alternative splicing, RNA editing, non-canonical splice sites, novel transcripts and the sequences of the 5′ UTR and 3′ UTR regions. The total coverage of reads along the genes was evenly distributed except for the ultimate 5′ and 3′ ends indicating that overall our RNA-Seq data are of high quality [34]. Although overall the read coverage was evenly distributed over genes, for individual genes the coverage was not evenly distributed; this phenomenon has also been reported in other RNA-Seq studies [32,33]. Interestingly, for nearly all genes the read coverage pattern between wt PH-1 and mutant *ebr1* is very similar. This suggests that each gene has a characteristic RNA-Seq profile, which could be related to secondary structure of particular domains of RNA molecules that interfere with RNA shearing and subsequent sequencing.

The background reads in RNA-Seq data sets have been reported to be low. For example in RNA-Seq data obtained from yeast, no reads matching a 3.5-kb deleted region were obtained, and very few reads matching to nontranscribed centromeres were identified [32]. A similar result was found in our RNA-Seq analysis; a comparison of the *EBR1* expression level between PH-1 and *ebr1* showed no transcription of the *EBR1* gene in the *ebr1* deletion mutant. In addition, we found that RNA-Seq data analysis for both PH-1 and *ebr1* RNA-Seq is very reproducible.

Analysis of the read distribution suggests that 12.9% of reads matched to intergenic regions, which is relatively high in comparison to 3% and 5% found in *H. sapiens* and *A. thaliana,* respectively [13,31]. This high percentage may at least partly reflect the lower quality of gene model prediction in *F. graminearum* compared to *H. sapiens* and *A. thaliana*. In the latter two genomes, several rounds of gene annotation have been performed and more experimental evidence has been provided to support the gene models. In addition, 16% of the reads could not be matched to a single location in the genome, a finding that was also reported in other species [13,31]. For instance, in *H. sapiens*, 20% of the intergenic reads match to multiple locations in the genome, of which 6% match to 2–10 locations and 14% to more than 10 locations [31]. Furthermore, we identified that most of the reads mapping to multiple locations originate from intergenic regions and UTRs, whereas only very few reads matched to coding regions, which suggests that the reads matching to each transcript are very specific and the read coverage of each transcript is a reliable reflection of the gene expression level.

RNA-Seq has been widely used to identify incorrect gene models and alternative splicing in different organisms [10,13,30,33]. However, to distinguish incorrect gene models from alternative splicing is a challenging and laborious task. In this study, all selected genes were manually examined in the CLC software package to identify reads showing splice sites. RT-PCR analysis on the selected genes confirmed that identification of incorrectly annotated gene models and alternative splicing appears reliable. In total 655 genes were identified with incorrect gene models in the Broad *F. graminearum* database. Excluding genes with no detectable expression or with low read coverage (less than 50 reads), the fraction of incorrect gene models in the published annotation of the Broad *F. graminearum* database is 10.3%. Gene model predictions in the MIPS *F. graminearum* database were considered to be of higher quality than those in the Broad *F. graminearum* database [8], which was confirmed by our RNA-Seq analysis. Nonetheless we could still improve many gene models predicted in the MIPS *F. graminearum* database. Even some of the manually revised gene models in the MIPS *F. graminearum* database appeared to be incorrect, indicating that gene annotations in the *F. graminearum* database still need to be improved and that RNA-Seq analysis can significantly improve the published gene models. In this study, RNA-Seq data were generated from mycelia growing in nutrient-rich medium. To investigate whether the incorrectly annotated genes are caused by alternative splicing, we also analyzed the available EST data generated from other conditions, such as carbon- and nitrogen- starved media and cultures of maturing perithecia [43]. These EST data support our discoveries that genes are incorrectly annotated, but six genes were identified that have two different transcripts, indicating that they might be alternatively spliced. Consequently, some of the genes classified in this study as incorrectly annotated genes might in fact be alternative spliced genes.

Alternative splicing has been investigated in many organisms including *H. sapiens*, *Caenorhabditis elegans*, *A. thaliana* and *C. neoformans*[13,37,44]. In *H. sapiens*, 95% of the genes undergo alternative splicing [11,44]; in *A. thaliana*, alternative splicing is estimated to be 42% [13]. In fungi much lower percentages of alternative splicing have been predicted, including 4.3% (277 genes) in *C. neoformans*, 1.3% (162 genes) in *A. flavus* and 1.4% (151 genes) in *M. grisea*[26,29]. We found alternative splicing in 231 genes (1.7%) in *F. graminearum*, but it should be noted that we have only analyzed expression in one growth condition and as fungi can adapt to many different environmental conditions we expect that this percentage will increase when transcription profiles under more different growth conditions are analyzed. At least 4 different types of alternative splicing exist in *F. graminearum*, of which intron retention appeared most prevalent, which is also the case in *A. thaliana*[13,45,46], whereas in *H. sapiens*, exon skipping is most prevalent [10].

In-frame analysis showed that the majority of the alternatively spliced transcripts identified in *F. graminearum* cause premature termination codons (PTCs), of which most are located in intronic regions. Also in *H. sapiens* and *A. thaliana*, a high percentage of alternatively spliced transcripts contain PTCs [13,47]. In *A. thaliana*, 77.9% of the alternatively spliced genes introduce PTCs and most of them are considered as potential targets of the nonsense mediated mRNA decay (NMD) [13,46]. NMD was initially identified in *S. cerevisiae* and later widely studied in higher eukaryotes [48-50], but so far, only a few studies on NMD are reported in filamentous fungi [51] and whether the PTCs identified in *F. graminearum* are also associated with NMD needs to be further investigated. Apart from PTC isoforms, some alternatively spliced transcripts encoding proteins with diverse length were identified. The effects of the diversity in length on the biological function of proteins are still unknown, but several functions including binding properties, intracellular localization, enzymatic activity or stability might be affected [52].

Alternative splicing appears widespread in eukaryotes, but the biological function of alternative splicing is still poorly understood. Some studies have shown that alternative splicing events are developmentally regulated or associated with the response to different environmental conditions [53,54]. For instance, in *A. thaliana*, the *CIRCADIAN CLOCK ASSOCIATED 1 (CCA1)* gene produces two different transcripts and their expression ratio is dependent of light and temperature [13]. Similarly, in *A. thaliana* splicing of serine/arginine-rich protein-encoding genes is altered in response to hormones or abiotic stresses [54]. In *H. sapiens*, a number of genes involved in apoptosis [55] and differentiation of embryonic stem cells are regulated by alternative splicing [19,56]. In our study, we have also demonstrated that for some genes the alternative splicing events are regulated at different vegetative growth stages in *F. graminearum*; their biological implications are not yet understood, but they might be important in adaptation of *F. graminearum* to changing external environmental conditions that occur during different growth stages.

As reported previously in other species, in addition to the canonical GT donor and AG acceptor sites in introns there are several non-canonical donor and acceptor sites, of which GC occurs most frequently as an alternative donor site [13,57,58]. The non-canonical splice sites in *F. graminearum* also showed that the GC donor, AG acceptor combination is prevalent, of which the proportion is consistent with what has been found in other organisms [13,58]. In addition, the nucleotide preferences flanking the GC donor splice site and AG acceptor splice site identified in *F. graminearum* are consistent with previous reports in other organisms [58].

## Conclusions

We have analyzed the transcriptome of *F. graminearum* during growth in nutrient-rich medium by RNA-Seq and identified transcripts of 84% of the predicted genes, which allowed us to not only significantly revise existing gene models present in the Broad *Fusarium* database but also to get preliminary information on the presence of alternative splicing in this fungus. This is one of the most comprehensive reports on alternative splicing in filamentous fungi. Our analyses indicate that the occurrence of alternative splicing in *F. graminearum* is lower than in *H. sapiens* and *A. thaliana*. Nevertheless, the expression of alternatively spliced genes appeared tightly regulated in different growth stage and can change from spore to mycelium within a few hours. This is the first indication that alternative splicing may be important in the developmental regulation in filamentous fungi. In the future, the biological functions of the different transcript isoforms and their encoded proteins need to be studied in more detail.

## Methods

### Fungal strains and culture conditions

*F. graminearum* isolates wt PH-1 (PH-1) and the mutant *ebr1* (*ebr1*) were used in this study. PH-1 is the sequenced strain [7] and *ebr1* is a knock out mutant derived from PH-1 and its phenotype was recently described [39]. To prepare the mycelium for RNA-Seq, both PH-1 isolate and *ebr1* were grown in liquid mung bean medium for 3 days to produce conidia (25°C, 200 rpm). Then 10^5^ conidia of PH-1 and *ebr1* were transferred to 400 ml liquid complete medium (CM) [59] and grown for 30 h to produce mycelium (25°C, 200 rpm).

### RNA isolation and RT-PCR

Mycelium harvested from PH-1 and *ebr1* was collected from liquid CM medium by filtration and ground in liquid nitrogen using a mortar and pestle. Ground mycelium was used for RNA extraction by TRIzol reagent (Invitrogen, Cat. No. 15596–018) according to the manufacturer’s instructions. The quality of RNA was evaluated by Agilent 2100.

For reverse transcription (RT)-PCR, isolated RNA was treated with DNase I (Fermentas, #EN0521) according to the manufacturer’s manual. The DNase I-treated RNA was reversely transcribed into cDNA by using M-MLV Reverse Transcriptase (Promega) according to the protocol described in the manual. cDNA was used as template to perform RT-PCR according to the following procedures: 20 μl reaction mixture including 2 μl 10 × reaction buffer, 0.8 μl dNTP (5 mM), 0.5 μl forward primer (10 μM), 0.5 μl reverse primer (10 μM), 1 μl template, 0.3 μl Taq DNA polymerase (Roche) and 14.9 μl ddH_2_O; reaction conditions including step 1 (94°C 4 min), step 2 (94°C 30s; 56°C 30s; 72°C 60s; this step was repeated 34 times) and step 3 (72°C 10 min). All primers used in this study are listed in Additional file 16.

### RNA-Seq analysis

Isolated RNA was enriched for mRNA by using oligo dT Dynabeads (Invitrogen) according to the manufacturer’s instructions and fragmented into fragments of 200–700 nucleotides by incubating at 70°C for 15 min in fragmentation buffer (Ambion). Fragmentation of mRNA was terminated by adding stop solution (Ambion) and used as template to synthesize the first strand cDNA by using random hexamers (Invitrogen). Subsequently dNTPs, RNase and DNA polymerase were added to the reaction solution to synthesize the second strand cDNA. The synthesized cDNA was purified by Qiaquick PCR kits and blunted by an End Repair reaction. Subsequently a single “A” base was added to the 3′ end of cDNA by using dATP and Klenow Exo Fragment. Later Illumina adaptors were linked to the cDNA ends. The adapted cDNA was run on agrose gel and ~200 bp cDNA fragments were selected. Finally, the cDNA was amplified and the obtained cDNA pool was subjected to high-throughput sequencing by Illumina HiSeq^TN^ 2000.

### Reads mapping

The gene database, the transcript database, the supercontig database and the UTR database of *F. graminearum* were downloaded from the Broad Institute (http://www.broadinstitute.org/annotation/genome/fusarium_group/MultiDownloads.html). All these databases along with RNA-Seq raw data were imported into the CLC genomic workbench software according to the method described in the manual. The “RNA-Seq analysis” option was used to map reads to each database at the following settings: minimum length fraction 0.9, minimum similarity fraction 0.8, and maximum number of hits for a read 30. The matched reads were visualized in the CLC interface.

### Identification of incorrect gene models and alternative splicing

Three strategies were employed to identify incorrect gene models and alternative splicing. In the first strategy, we mapped all reads from the PH-1 and *ebr1* RNA-Seq data (25,720,650 reads in total) against complete transcript database (only exonic regions). After this round of mapping, 13,073,825 unmapped reads were obtained that were subsequently aligned against the 5′ UTR (1000 bp) and 3′ UTR (1000 bp) databases for the second round of mapping, after which 6,995,901 unmapped reads were obtained. This set of unmapped reads can be divided in four fractions: (i) reads matching to intergenic regions, (ii) reads matching to intronic regions, (iii) reads matching to the border of coding regions and UTRs, and (iv) non-mapped reads. Finally, the 6,995,901 unmapped reads were aligned against the gene database (including exons, introns and UTRs). From this round of mapping, 732,254 reads were identified matching to genes, from which we could collect all genes with introns matched by reads. In the second strategy, all reads were mapped against the transcript database and the matched reads for each transcript were visualized in the CLC interface. We browsed all transcripts that contained more than 200 matched reads, from which we collected transcripts with non-matched or mismatched regions. For the non-matched regions, there must be at least one read flanking this region showing a splice site. In the third strategy, we employed the TopHat program to identify incorrect gene models and alternative splicing according to a previously described protocol [40,60].

All the genes collected by these three strategies were first examined in the MIPS *F. graminearum* database to exclude genes that had already been revised manually*.* The remaining genes were manually examined in the CLC software package by comparing RNA-Seq reads with the predicted gene models to identify incorrectly annotated genes or alternatively spliced genes. A number of genes from each category were selected for confirmation by RT-PCR.

### Identification of nTARs

We aligned all reads against the supercontigs of *F. graminearum* and collected all reads matched regions (more than two read coverage on average and more than 150 bp in length) that located in the intergenic regions 200 bp away from flanking gene models. To analyze whether these nTARs encode mRNAs, we collected their sequences and blasted them against the MIPS *F. graminearum* database to identify novel genes that had already been annotated and against the Broad *Fusarium* database to identify orthologous genes in *F. oxysporum* and *F. verticillioides*.

### RNA editing analysis

All reads from PH-1 and *ebr1* were first aligned against the gene database of *F. graminearum* in CLC and the “SNP analysis” module was used to analyze putative SNPs between the RNA-Seq and the genome data. To confirm SNPs, primers were designed in flanking regions and PCRs were performed by using genomic DNA and cDNA, respectively, as templates. The amplicons were sequenced and the obtained sequences were aligned to the annotated genomic sequence to identify putative RNA editing.

## Abbreviations

CM: Complete medium; nTARs: Novel transcriptional active regions; UTRs: Untranslated regions; SNPs: Single nucleotide polymorphisms.

## Competing interests

The authors declare that they have no competing interests.

## Authors’ contributions

CZ performed all experiments and bioinformatics analyses. CW, PJGM, DZ and TvdL supervised the project and revised the manuscript. All authors read and approved the final manuscript.

## Supplementary Material

Additional file 1 **Gene expression analysis in wt PH-1.** Expression analysis of the 13321 predicted genes in wt PH-1 after incubation in liquid CM medium for 30 h. Click here for file

Additional file 2 **Gene expression analysis in mutant *ebr1*.** Expression analysis of the 13321 predicted genes in mutant *ebr1* after incubation in liquid CM medium for 30 h. Click here for file

Additional file 3 PDF file containing all supplementary figures and their legends. Click here for file

Additional file 4 **Incorrectly annotated genes that have been revised in the MIPS *F. graminearum* database.** 278 genes that were incorrectly annotated in Broad *F. graminearum* database have already been revised in MIPS *F. graminearum* database. Click here for file

Additional file 5 **Genes with incorrect intron predictions.** 119 genes with incorrect intron predictions were identified and revised. Click here for file

Additional file 6 **Genes with novel introns.** 40 genes with novel introns were identified and revised. Click here for file

Additional file 7 **Genes with incorrect splice sites.** 164 genes (186 introns) with incorrectly predicted splice sites were identified and revised. Click here for file

Additional file 8 **Genes with intron retention.** 211 genes with intron retention were shown. Click here for file

Additional file 9 **Genes with alternative 5′ splicing.** 21 genes with alternative 5′ splicing were shown. Click here for file

Additional file 10 **Genes with alternative 3′ splicing.** 12 genes with alternative 3′ splicing were shown. Click here for file

Additional file 11 **Genes with exon skipping.** 4 genes with exon skipping were shown. Click here for file

Additional file 12 **Genes with non-canonical splice sites.** Non-canonical splice sites were identified in 28 genes. Click here for file

Additional file 13 **nTARs identified in intergenic regions.** 2459 nTARs were identified in intergenic regions. The expression levels of these nTARs and their orthologs in *F. oxysporum* and *F. verticillioides* were analyzed. Click here for file

Additional file 14 **Identification of 5′ UTRs.** 5′ UTRs of 5951 genes were determined by RNA-Seq data. Click here for file

Additional file 15 **Identification of 3′ UTRs.** 3′ UTRs of 6405 genes were determined by RNA-Seq data. Click here for file

Additional file 16 Primers used in this study. Click here for file
