# Supplementary material for: RNA-Seq analysis reveals new gene models and alternative splicing in the fungal pathogen Fusarium graminearum
Source: BMC Genomics. 2013 Jan 16;14:21. doi: 10.1186/1471-2164-14-21 (PMC3577648; doi:10.1186/1471-2164-14-21)
Supplement: Additional file 3 — PDF file containing all supplementary figures and their legends. [file 1471-2164-14-21-S3.pdf]

Supplementary Figures

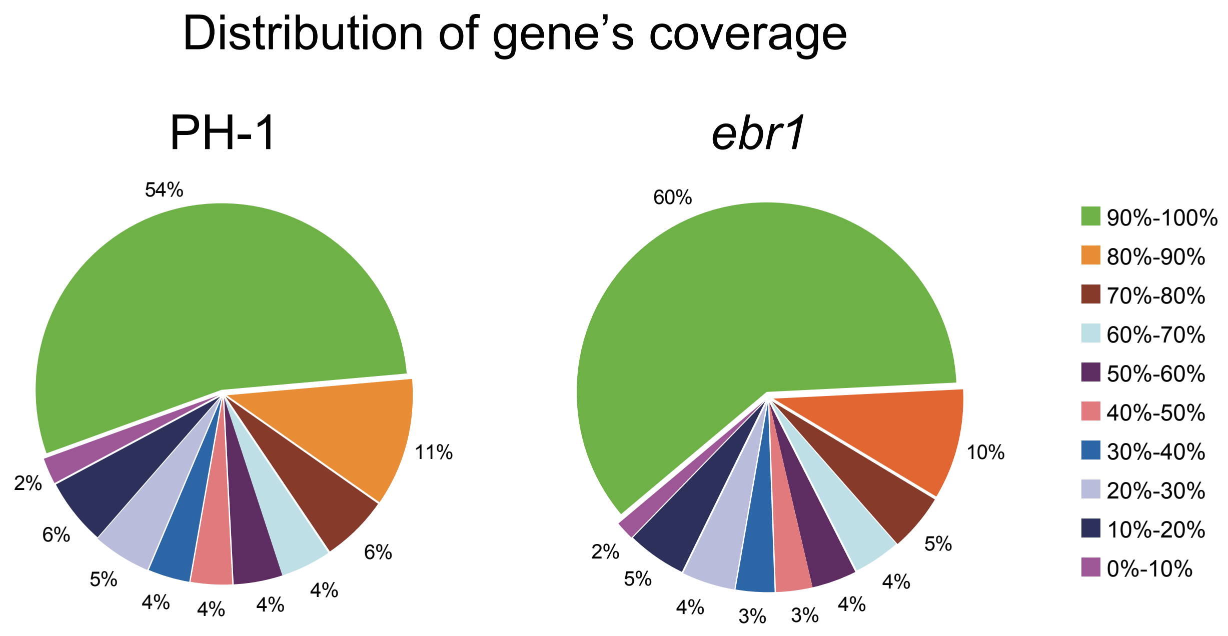

Figure S1. Statistical analysis of gene coverage by RNA-seq reads in wt PH-1 and mutant *ebr1*. Graphs show that 54% of the genes in PH-1 and 60% of the genes in *ebr1* are over 90% covered by reads.

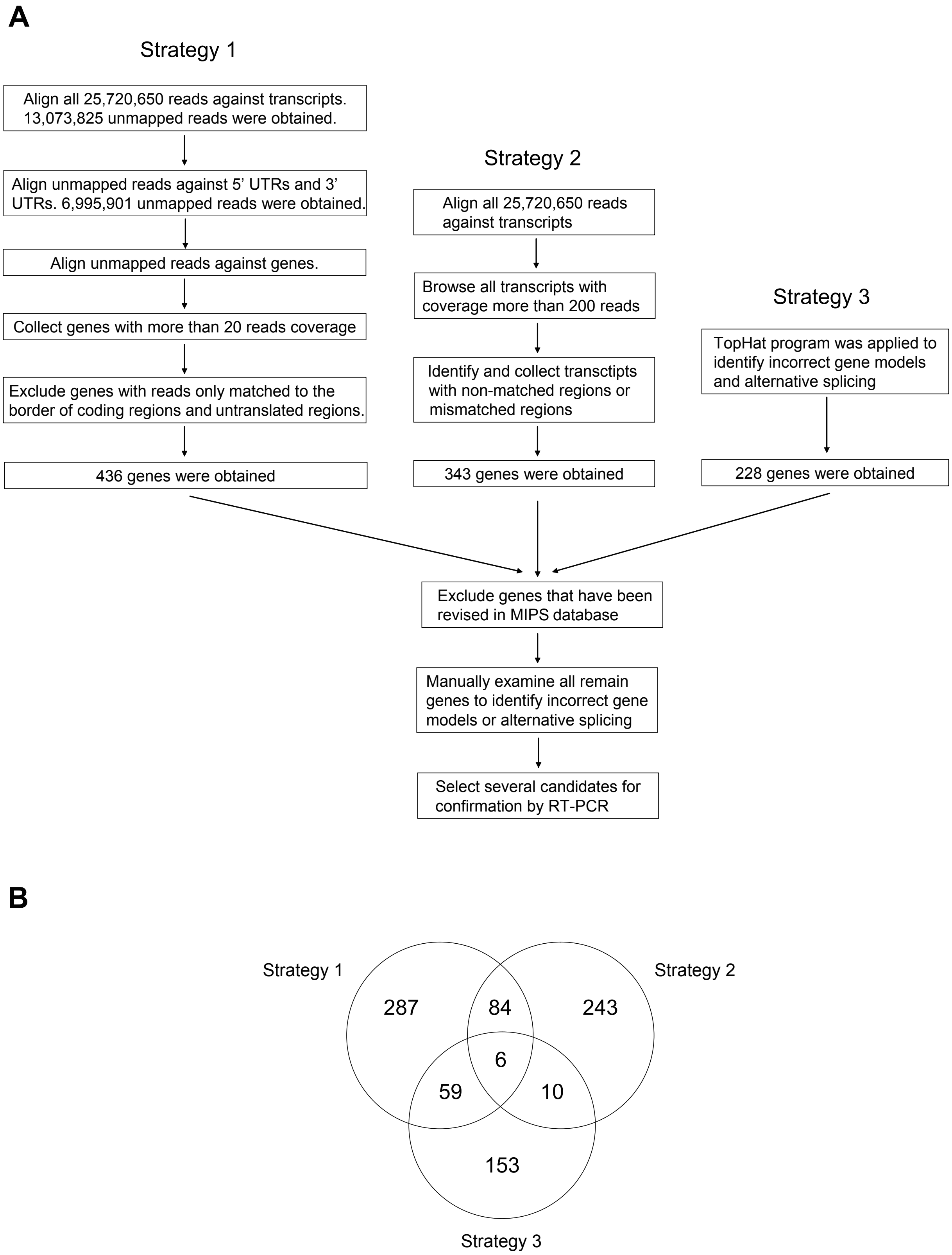

Figure S2. Strategies used to identify incorrect gene models and alternative splicing. A. Three complementary strategies were employed to identify genes with incorrect gene models or alternative splicing. **B.** Comparison of genes identified by three different strategies.

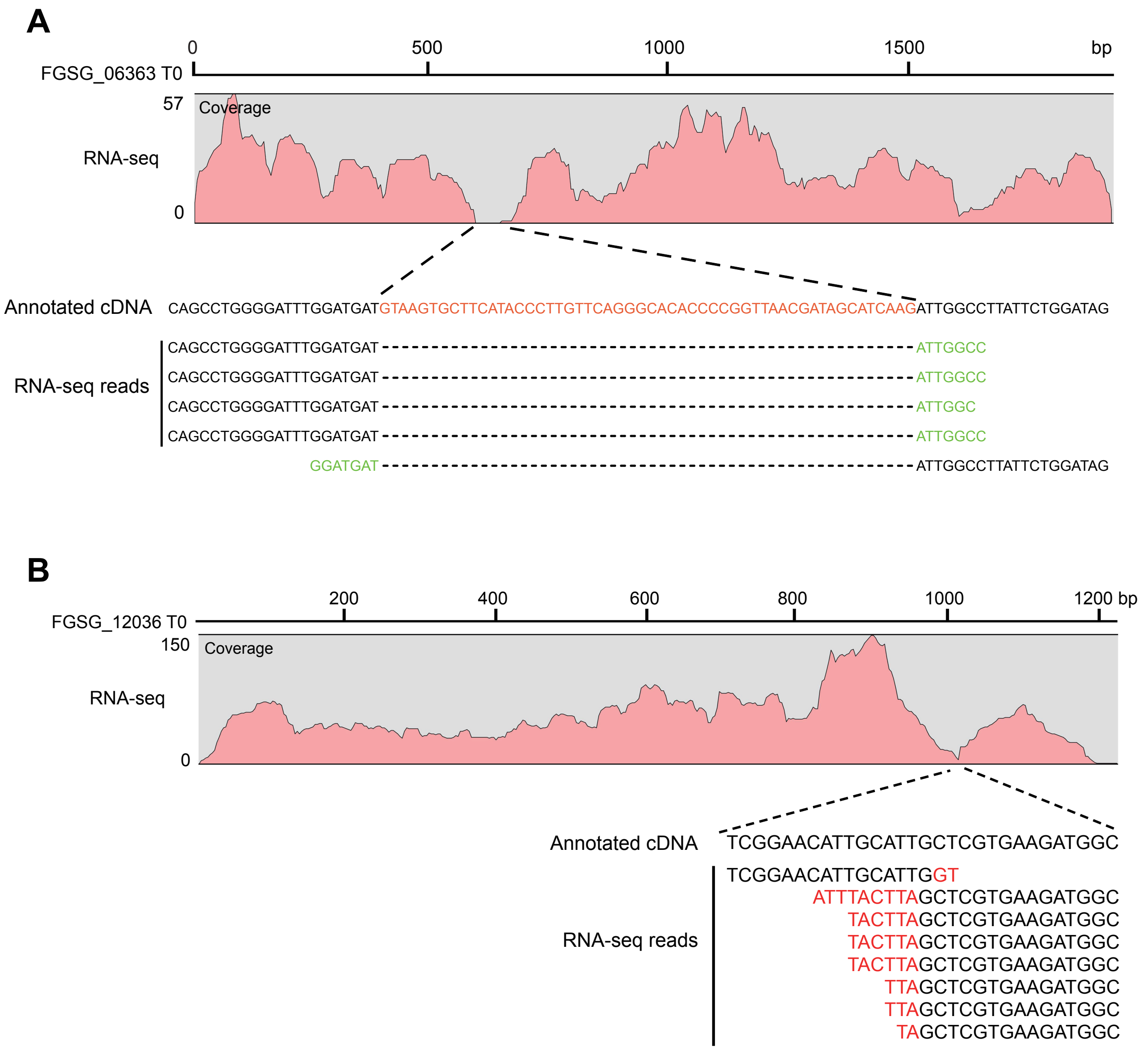

Figure S3. Two examples of transcripts derived from incorrect gene models. A. Transcript with non-matched region. When aligning reads with the annotated transcript of gene FGSG_06363, a non-matched region was identified. Five reads flanking this region show splice sites (green letters), indicative of a novel intron in gene FGSG_06363. **B.** Transcript with mismatched region. One position with mismatched reads was identified when aligning reads with the annotated transcript of gene FGSG_12036. The nucleotides (red letters) at the end of eight reads do not match to the transcript of FGSG_12036, indicative of an incorrectly predicted intron in gene FGSG_12036. Black letters represent exonic region; orange letters represent intronic region.

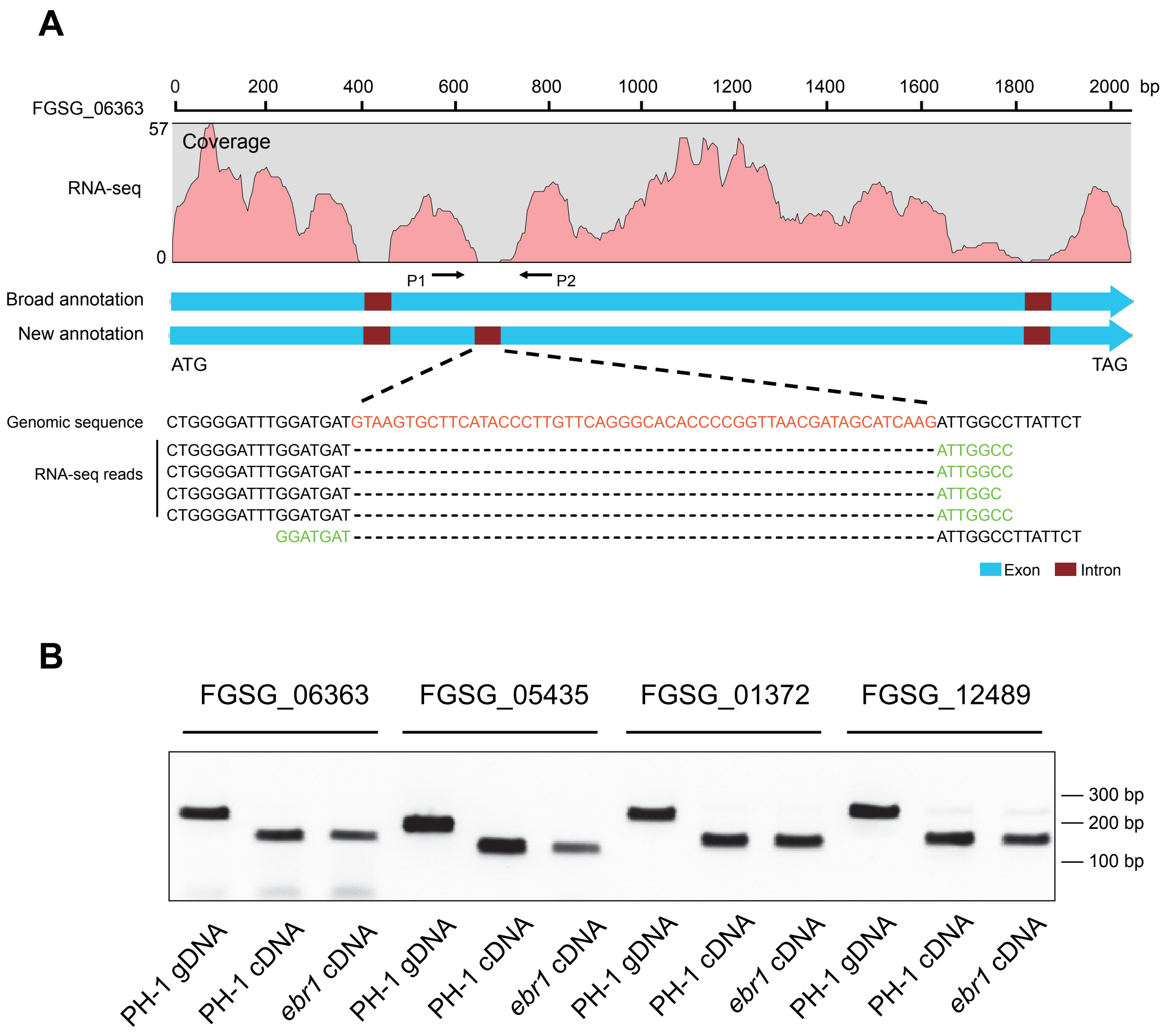

Figure S4. Identification of novel introns. A. A novel intron was identified in gene FGSG_06363. The novel intron was supported by the reads that show the intron splice sites (green letters). Black letters represent exonic region; orange letters represent the intronic region. **B.** Four genes were randomly selected for the confirmation of novel introns Primers were designed flanking the intronic regions as shown in A.

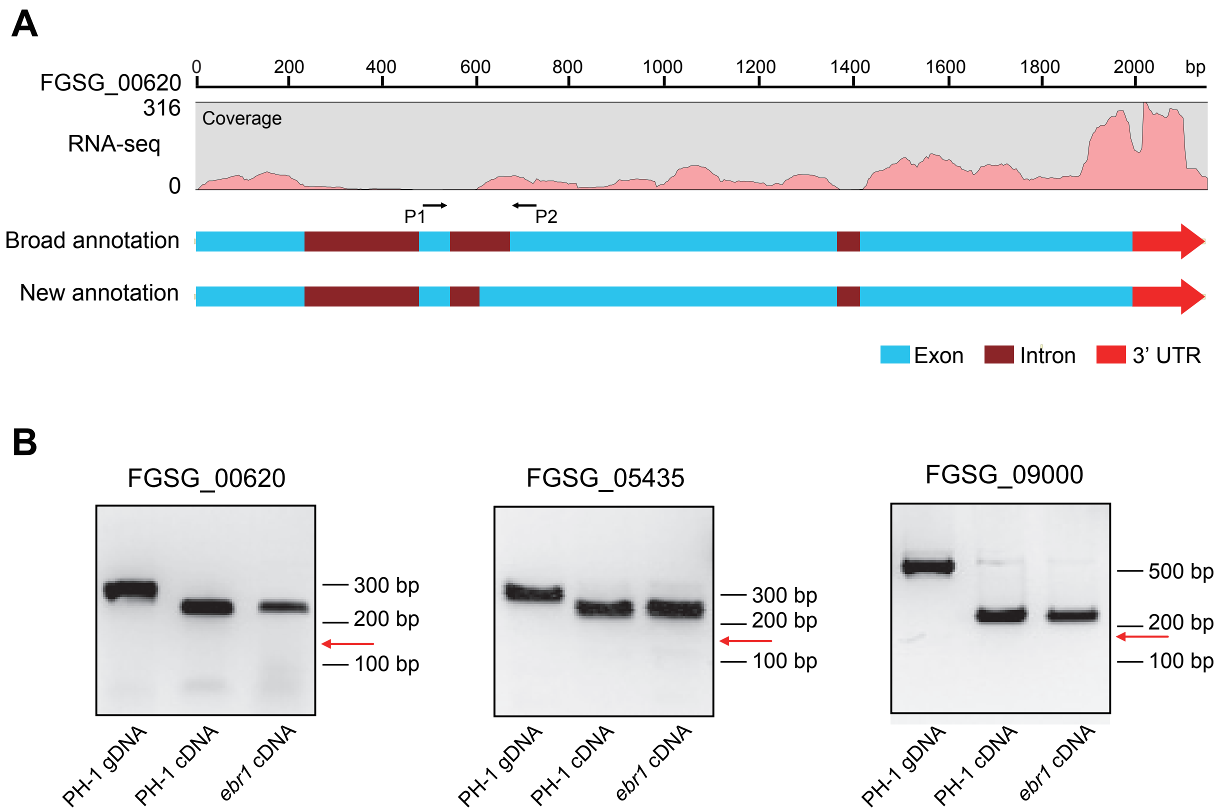

Figure S5. Identification of incorrect intron splice sites. A. RNA-seq data show that the 3' splice site of the second intron of gene FGSG_00620 is different from the annotated gene present in the Broad *F. graminearum* database. **B.** Three genes were selected to confirm the incorrect intron splice sites. Red arrows indicate the size of the amplified products by PCR that should be obtained according to gene models present in the Broad *F. graminearum* database. Primers were designed flanking the intronic regions.

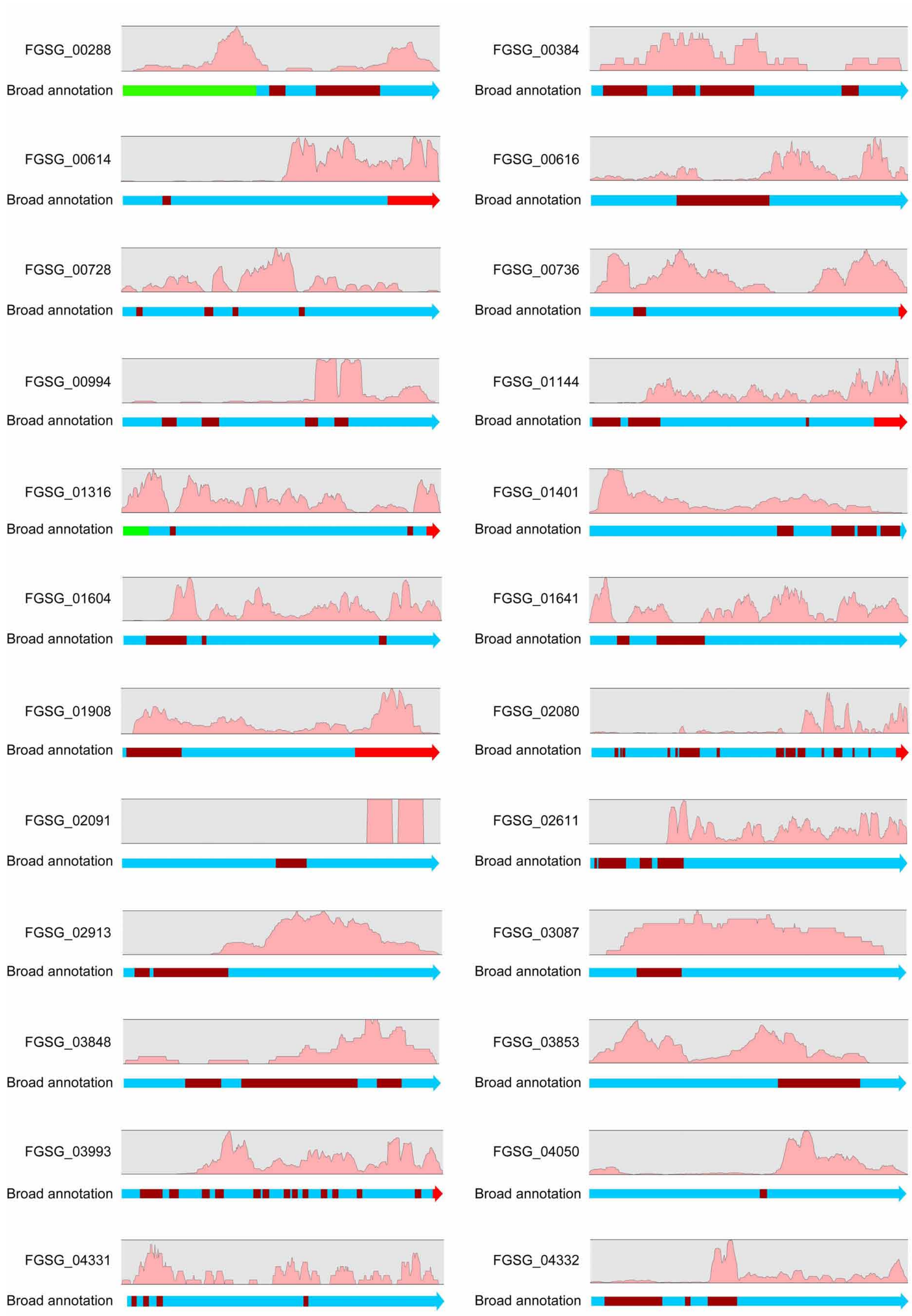
FGSG_00288
Broad annotation
FGSG_00384
Broad annotation
FGSG_00614
Broad annotation
FGSG_00616
Broad annotation
FGSG_00728
Broad annotation
FGSG_00736
Broad annotation
FGSG_00994
Broad annotation
FGSG_01144
Broad annotation
FGSG_01316
Broad annotation
FGSG_01401
Broad annotation
FGSG_01604
Broad annotation
FGSG_01641
Broad annotation
FGSG_01908
Broad annotation
FGSG_02080
Broad annotation
FGSG_02091
Broad annotation
FGSG_02611
Broad annotation
FGSG_02913
Broad annotation
FGSG_03087
Broad annotation
FGSG_03848
Broad annotation
FGSG_03853
Broad annotation
FGSG_03993
Broad annotation
FGSG_04050
Broad annotation
FGSG_04331
Broad annotation
FGSG_04332
Broad annotation

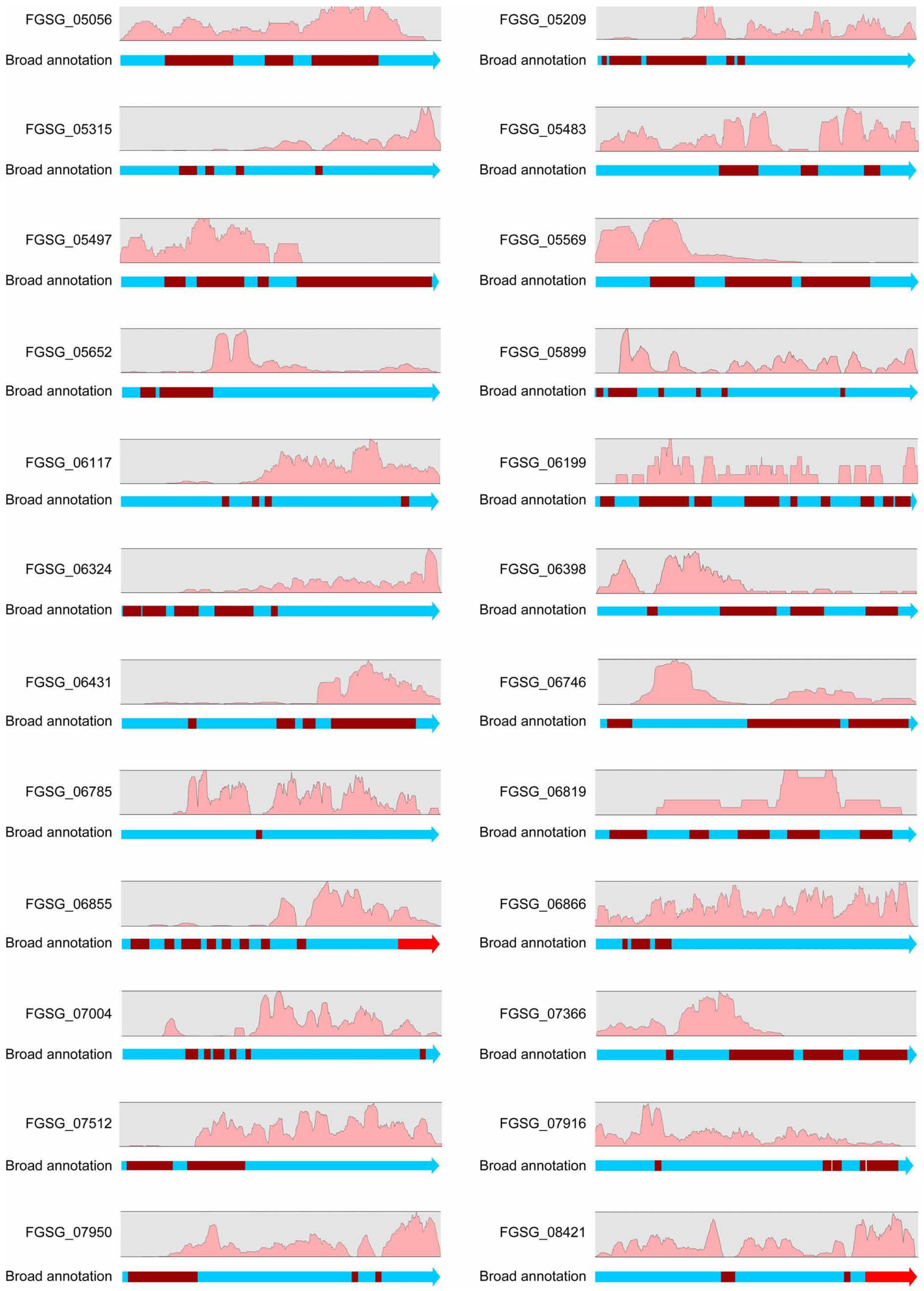
FGSG_05056
Broad annotation
FGSG_05209
Broad annotation
FGSG_05315
Broad annotation
FGSG_05483
Broad annotation
FGSG_05497
Broad annotation
FGSG_05569
Broad annotation
FGSG_05652
Broad annotation
FGSG_05899
Broad annotation
FGSG_06117
Broad annotation
FGSG_06199
Broad annotation
FGSG_06324
Broad annotation
FGSG_06398
Broad annotation
FGSG_06431
Broad annotation
FGSG_06746
Broad annotation
FGSG_06785
Broad annotation
FGSG_06819
Broad annotation
FGSG_06855
Broad annotation
FGSG_06866
Broad annotation
FGSG_07004
Broad annotation
FGSG_07366
Broad annotation
FGSG_07512
Broad annotation
FGSG_07916
Broad annotation
FGSG_07950
Broad annotation
FGSG_08421
Broad annotation

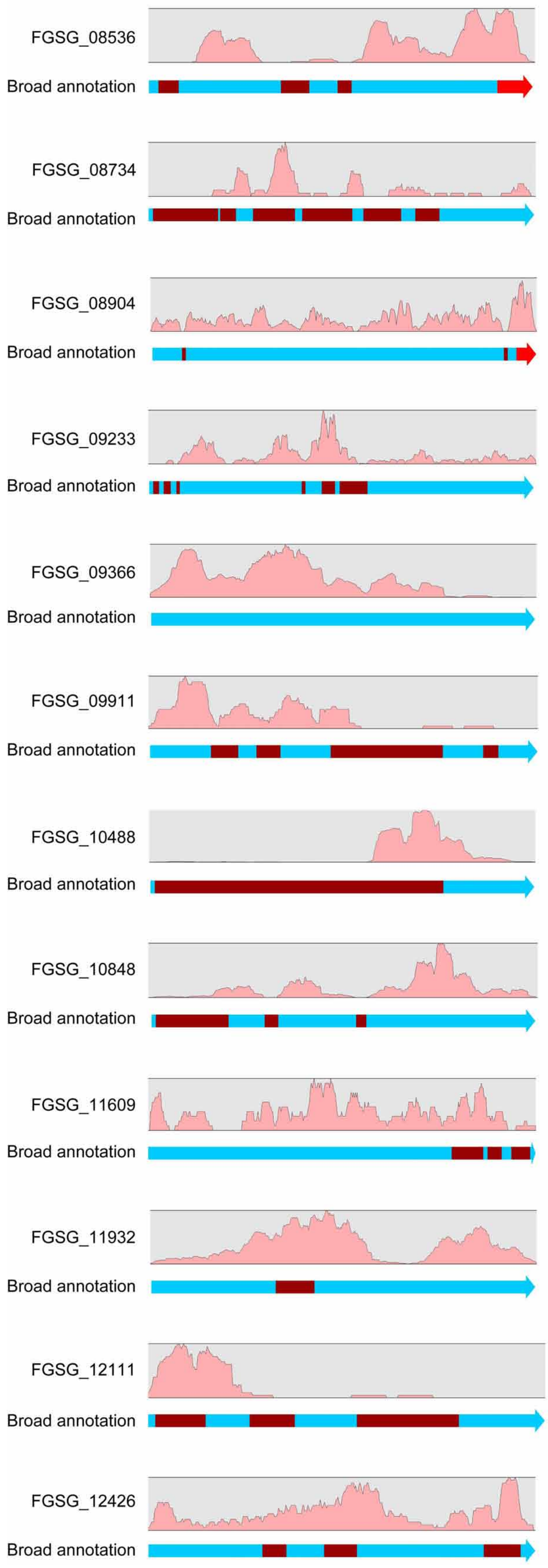
FGSG_08536
Broad annotation
FGSG_08698
Broad annotation
FGSG_08734
Broad annotation
FGSG_08790
Broad annotation
FGSG_08904
Broad annotation
FGSG_09068
Broad annotation
FGSG_09233
Broad annotation
FGSG_09275
Broad annotation
FGSG_09366
Broad annotation
FGSG_09708
Broad annotation
FGSG_09911
Broad annotation
FGSG_10051
Broad annotation
FGSG_10488
Broad annotation
FGSG_10786
Broad annotation
FGSG_10848
Broad annotation
FGSG_10877
Broad annotation
FGSG_11609
Broad annotation
FGSG_11881
Broad annotation
FGSG_11932
Broad annotation
FGSG_12039
Broad annotation
FGSG_12111
Broad annotation
FGSG_12159
Broad annotation
FGSG_12426
Broad annotation
FGSG_12660
Broad annotation

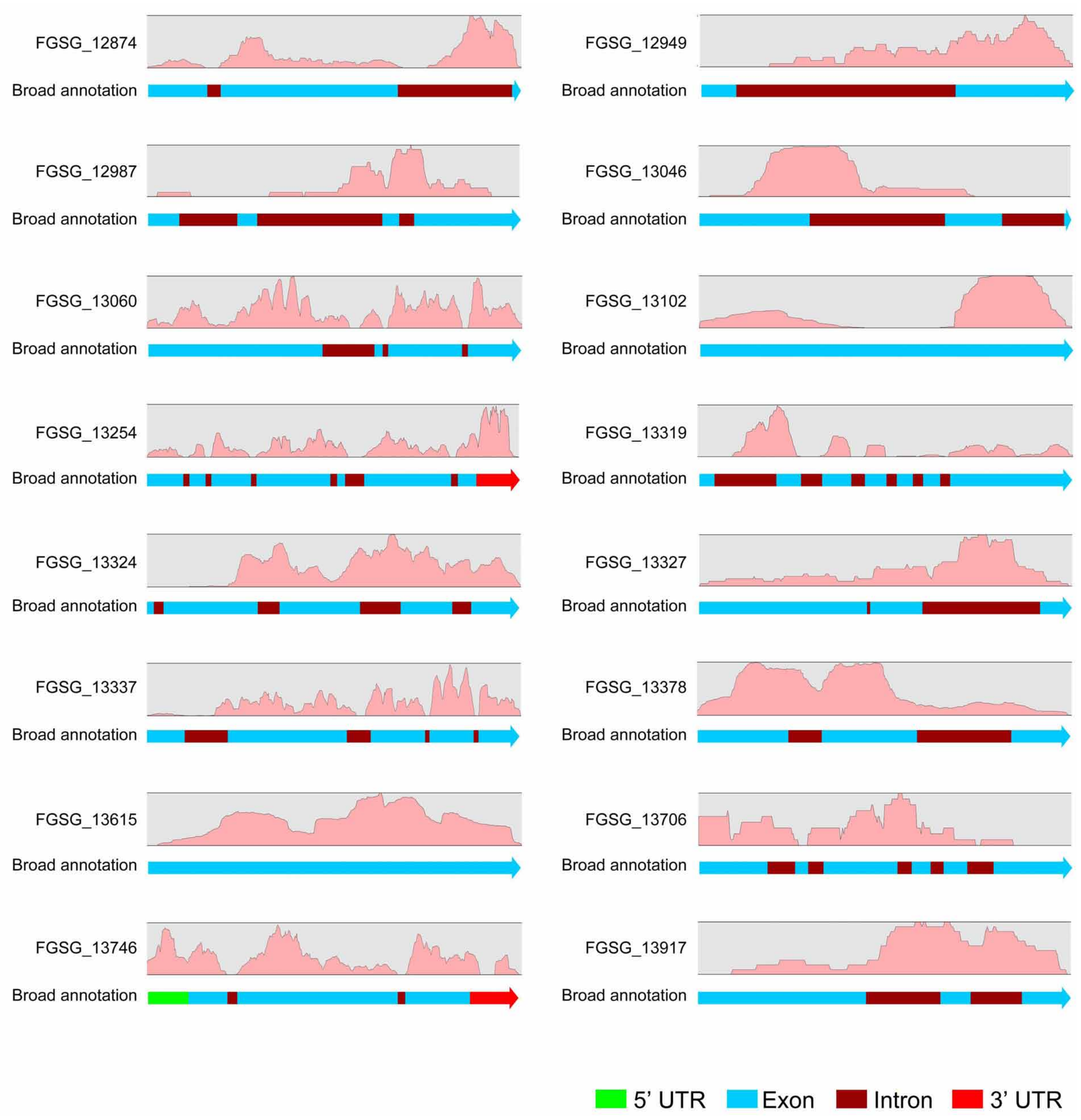

Figure S6. Comparison of RNA-seq data with other incorrect gene models. 88 incorrect gene models were identified but intron splice sites in these genes are still unclear. Comparison of RNA-seq data with these predicted gene models are shown.

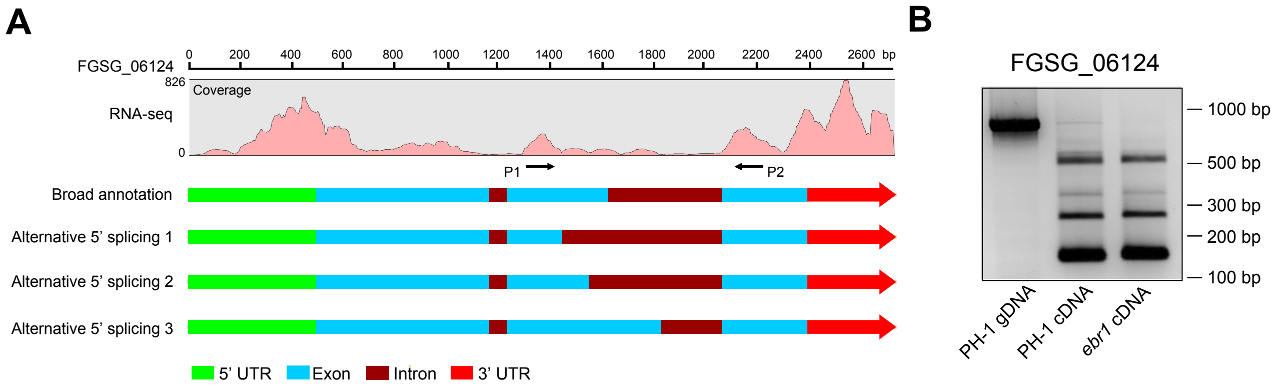

C

```
                                                                              Transmembrane regions
Broad annotation           MASSDIKHDYSMEAGLLNTDREERIMSSKEALLQDSESAQITPRTTTKRISPAGKALIALAACCLLGAGVASIGGATRFC   aa 80
Alternative 5' splicing 1  MASSDIKHDYSMEAGLLNTDREERIMSSKEALLQDSESAQITPRTTTKRISPAGKALIALAACCLLGAGVASIGGATRFC   aa 80
Alternative 5' splicing 2  MASSDIKHDYSMEAGLLNTDREERIMSSKEALLQDSESAQITPRTTTKRISPAGKALIALAACCLLGAGVASIGGATRFC   aa 80
Alternative 5' splicing 3  MASSDIKHDYSMEAGLLNTDREERIMSSKEALLQDSESAQITPRTTTKRISPAGKALIALAACCLLGAGVASIGGATRFC   aa 80

Broad annotation           PGHLRHGDLVGKHGMSGHRANLSESMAQMVRRQNTPAPTDTEDSTTEPTNAVDEASNEASAPTAPTAPTAPTAPTAPTAP   aa 160
Alternative 5' splicing 1  PGHLRHGDLVGKHGMSGHRANLSESMAQMVRRQNTPAPTDTEDSTTEPTNAVDEASNEASAPTAPTAPTAPTAPTAPTAP   aa 160
Alternative 5' splicing 2  PGHLRHGDLVGKHGMSGHRANLSESMAQMVRRQNTPAPTDTEDSTTEPTNAVDEASNEASAPTAPTAPTAPTAPTAPTAP   aa 160
Alternative 5' splicing 3  PGHLRHGDLVGKHGMSGHRANLSESMAQMVRRQNTPAPTDTEDSTTEPTNAVDEASNEASAPTAPTAPTAPTAPTAPTAP   aa 160

                                                       Prolipoprotein diacylglyceryl transferase
Broad annotation           TAPTAPTAPSDTAVPPSDTAQAPTDQASATQAEPSDEPSDAPTNAASETQSQASVTQKPSDTSRPSATAAQSTEEDSATE   aa 240
Alternative 5' splicing 1  TAPTAPTAPSDTAVPPSDTAQAPTDQASATQAEPSDEPSDAPTNAASETQSQASVTQKPSDTSRPSATAAQSTEEDSATE   aa 240
Alternative 5' splicing 2  TAPTAPTAPSDTAVPPSDTAQAPTDQASATQAEPSDEPSDAPTNAASETQSQASVTQKPSDTSRPSATAAQSTEEDSATE   aa 240
Alternative 5' splicing 3  TAPTAPTAPSDTAVPPSDTAQAPTDQASATQAEPSDEPSDAPTNAASETQSQASVTQKPSDTSRPSATAAQSTEEDSATE   aa 240

Broad annotation           DTPSATPSDEEPTKPASNTASTQGTTLRTTETRPASTQSDPESTADDSATATGTSDSEEEPSETALSSRASTSNPPAPSA   aa 320
Alternative 5' splicing 1  DTPSATPSDEEPTKPASNTASTQGTTLRTTETRPASTQSDPESTADDSATAT----------------------------   aa 292
Alternative 5' splicing 2  DTPSATPSDEEPTKPASNTASTQGTTLRTTETRPASTQSDPESTADDSATATGTSDSEEEPSETALSSRASTSNPPAPSA   aa 320
Alternative 5' splicing 3  DTPSATPSDEEPTKPASNTASTQGTTLRTTETRPASTQSDPESTADDSATATGTSDSEEEPSETALSSRASTSNPPAPSA   aa 320

Broad annotation           TTSTAASTSTRTNSLPDTSSFSELPSETVSS-------------------------------------------------   aa 351
Alternative 5' splicing 1  --------------------------------------------------------------------------------   aa 292
Alternative 5' splicing 2  TTSTAA--------------------------------------------------------------------------   aa 326
Alternative 5' splicing 3  TTSTAASTSTRTNSLPDTSSFSELPSETVSSGETNQSTVLDETSTAADASTDVDTATVLSSTTDDASDVLSDLTSEPASK   aa 400

Broad annotation           -------------------DDQETATQTDEATQTDEDKTTTETTESERRTITGTGTKPTSSAIPKTFTSTLEDGGVTTLT   aa 412
Alternative 5' splicing 1  -------------------DDQETATQTDEATQTDEDKTTTETTESERRTITGTGTKPTSSAIPKTFTSTLEDGGVTTLT   aa 353
Alternative 5' splicing 2  -------------------DDQETATQTDEATQTDEDKTTTETTESERRTITGTGTKPTSSAIPKTFTSTLEDGGVTTLT   aa 387
Alternative 5' splicing 3  STQESGTTSRTSEGRETTTDDQETATQTDEATQTDEDKTTTETTESERRTITGTGTKPTSSAIPKTFTSTLEDGGVTTLT   aa 480

Broad annotation           STSWVEVVPAAEATGSSNPDLQNAAPRSVGSILGAVVGMVFGGVILL                                    aa 459
Alternative 5' splicing 1  STSWVEVVPAAEATGSSNPDLQNAAPRSVGSILGAVVGMVFGGVILL                                    aa 400
Alternative 5' splicing 2  STSWVEVVPAAEATGSSNPDLQNAAPRSVGSILGAVVGMVFGGVILL                                    aa 434
Alternative 5' splicing 3  STSWVEVVPAAEATGSSNPDLQNAAPRSVGSILGAVVGMVFGGVILL                                    aa 527
```

Figure S7. Example of alternative 5' splicing. A. RNA-seq reads show that there are four different 5' splice sites of the second intron in gene FGSG_06124. **B.** All four transcripts with different sizes were confirmed by RT-PCR. Primers were designed flanking the intronic region as shown in A. **C.** Protein alignment shows the consequence of the alternative 5' splicing in gene FGSG_06124. Several amino acids are present or absent in the alternative proteins compared to the annotated protein.

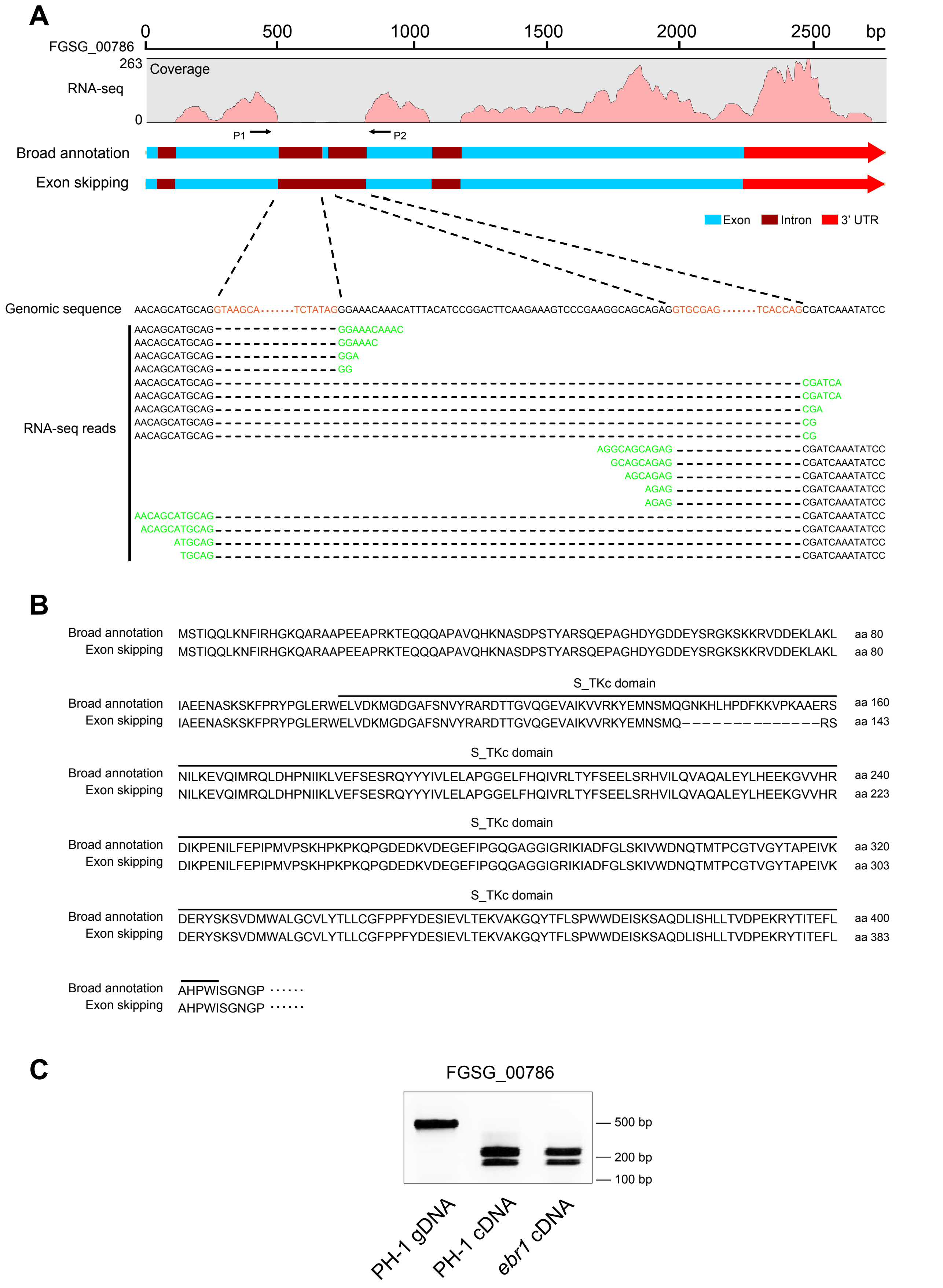

Figure S8. Example of exon skipping. A. RNA-seq data show that the third exon is skipped in gene FGSG_00786. From the reads on the left side, some intron splice sites (green letters) match to the third exon and some to the fourth exon. Similarly, the splice sites from the reads on the right side match to either the third or the second exon. **B.** Protein alignment shows that 17 amino acids are absent in the S_TKc domain in case of exon skipping. **C.** RT-PCR confirmed exon skipping of the third exon by detection of two bands with the expected sizes.

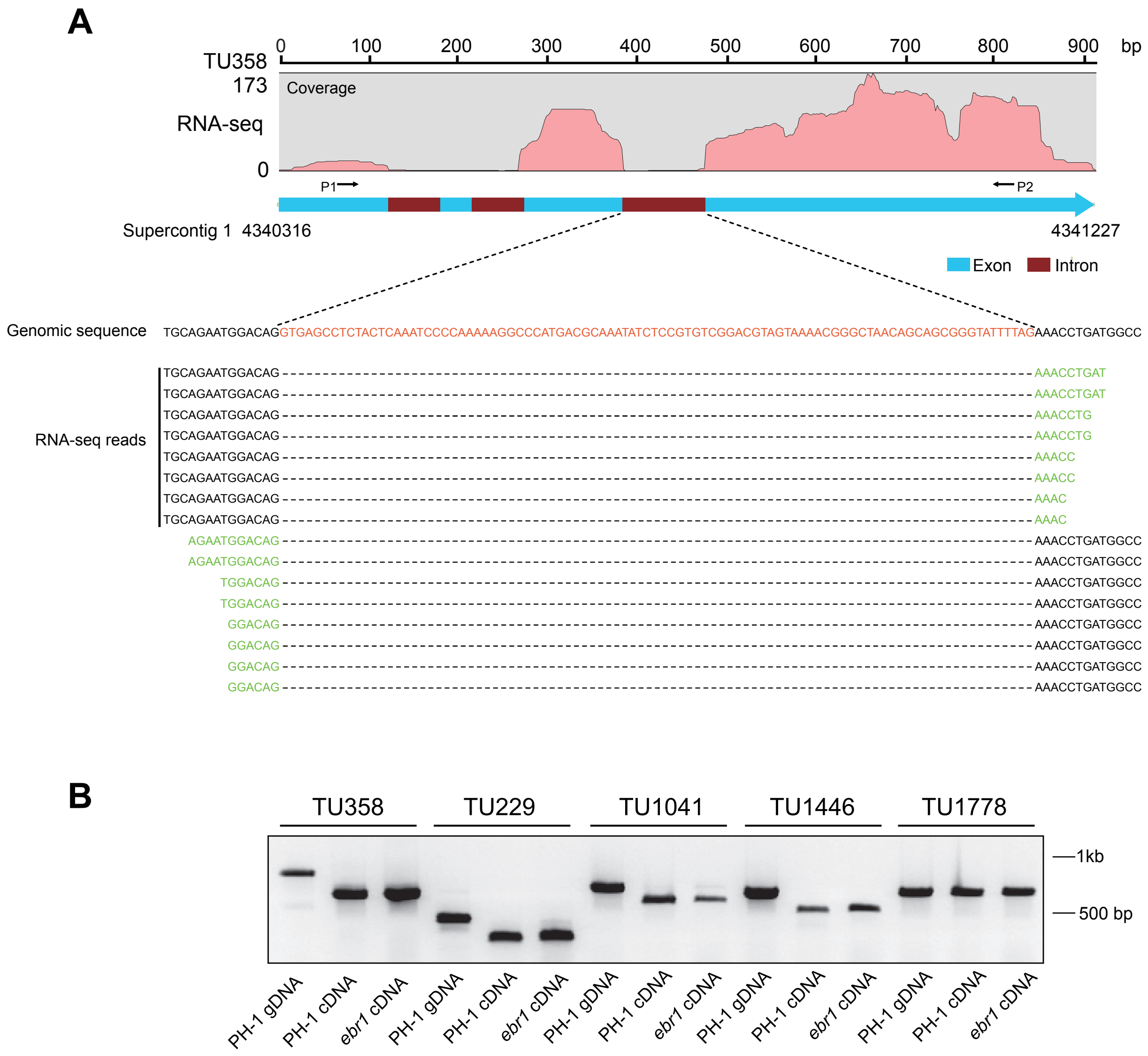

Figure S9. Identification of novel transcriptional active regions (nTARs). A. TU358 is shown as one of the nTARs encoding a putative mRNA. Three introns in TU358 were identified and one of them is displayed. **B.** Four nTARs containing intronic regions and one nTARs without intronic region were selected for confirmation by RT-PCR.

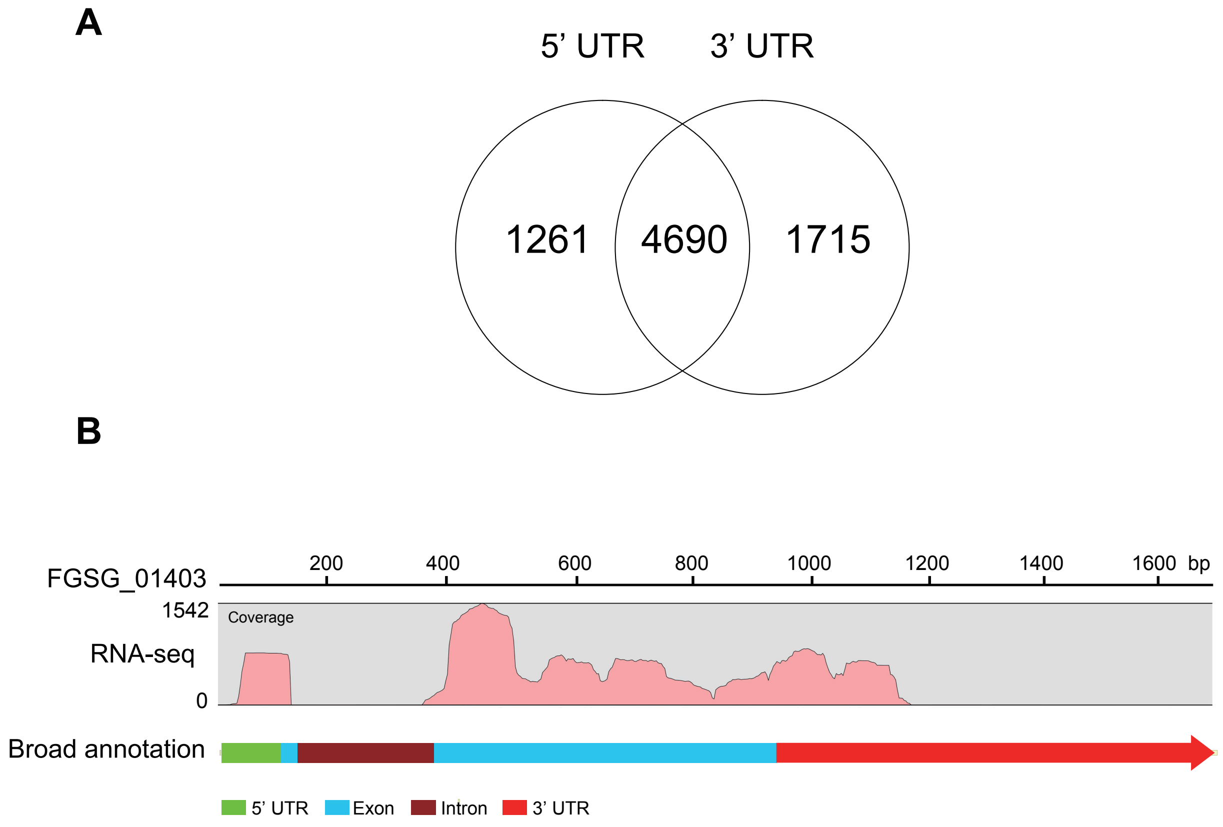

Figure S10. Determination of 5’ and 3’ UTR regions. A. For 4690 genes, both 5’ UTRs and 3’ UTRs were identified. For 1261 genes only 5’ UTRs and for 1715 genes only 3’ UTRs were identified. **B.** Comparison of UTRs predicted in the Broad *F. graminearum* database to RNA-seq data identified some incorrectly predicted UTRs. The graph shows one example where the length of the 3’ UTR in gene FGSG_01403 is shorter than the length annotated in the Broad *F. graminearum* database.
